# The association between agricultural activities and arthritis in middle-aged and elderly people: Findings from a cohort study based on CHARLS

**DOI:** 10.1371/journal.pone.0326447

**Published:** 2025-06-26

**Authors:** Xufeng Wang, Guoqiang Zou, Wenqi Zhang, Yi Zhang, Rongwei Zheng, Shufeng Li

**Affiliations:** 1 The First Clinical Medical College, Shandong University of Traditional Chinese Medicine (SDUTCM), Jinan, Shandong Province, China; 2 Department of Orthopedic Surgery, The First Affiliated Hospital of Shandong First Medical University & Shandong Provincial Qianfoshan Hospital, Shandong Key Laboratory of Rheumatic Disease and Translational Medicine, Jinan, Shandong Province, China; Shanghai Jiaotong University: Shanghai Jiao Tong University, CHINA

## Abstract

The number of arthritis samples in China has been increasing. Currently, there is limited research on the relationship between agricultural activities and arthritis. This study aimed to investigate the correlation between agricultural activities and arthritis risk based on the China Health and Retirement Longitudinal Study (CHARLS). A total of 694 participants from the 2015 CHARLS study were included, with 443 samples as controls and 251 samples classified as affected. Baseline characteristics of all participants were compared using the Student t-test and Chi-square test. Subsequently, the association between agricultural activities and arthritis risk was explored through multivariable generalized linear models (GLM) and weighted logistic regression models. Additionally, the diagnostic performance and clinical utility of agricultural activities for arthritis were evaluated using receiver operating characteristic (ROC) curves and decision curve analysis. Finally, the difference in model prediction performance before and after adjusting for covariates was assessed using the net reclassification index (NRI) and integrated discrimination improvement (IDI). Five covariates showed significant associations with arthritis, and agricultural activities had a significant effect (P = 0.026). Furthermore, a significant positive correlation was observed between agricultural activities and arthritis (Model 1: odds ratio (OR)=1.44, 95% confidence interval (95%CI): 1.06–1.97, P = 0.021; Model 2: OR=1.61, 95%CI: 1.17–2.24, P = 0.004; Model 3: OR=1.74, 95%CI: 1.17–2.60, P = 0.007). Risk stratification analysis further indicated that agricultural activities were a risk factor for arthritis (OR=1.736, 95%CI: 1.168–2.597, P < 0.01). Moreover, after adjusting for covariates, ROC curve analysis and decision curve analysis demonstrated good predictive performance of agricultural activities for arthritis. Lastly, the NRI and IDI indices indicated that Model 3 outperformed Models 1 and 2 in prediction performance. In conclusion, a significant positive correlation existed between agricultural activities and arthritis risk, providing insights for the early detection and prevention of arthritis.

## 1. Introduction

Arthritis is a prevalent condition among middle-aged and elderly people, affecting joints and surrounding tissues due to inflammatory processes triggered by factors such as inflammation, infection, degeneration, trauma, or others [1–4]. It encompasses hundreds of distinct conditions, characterized primarily by pain, restricted mobility, and joint deformities. Osteoarthritis (OA) and rheumatoid arthritis (RA) are the most prevalent types among more than 100 variants. Arthritis is an important public health issue and the second most common disability diseases in the world. Studies have shown that prevalence varies by race and geography [5,6]. Worldwide, the prevalence of OA cases increased by 113.25% [7,8]. According to statistics, there are currently more than 100 million arthritis patients in China, and half of the people aged 50 and above suffer from OA, and the number is growing steadily [7,9]. Arthritis not only leads to health consequences such as joint damage, pain, and mobility disorders, but also places a burden on multiple physiological systems such as cardiovascular, renal, and neurological systems. Severe complications can even increase mortality rates [10]. Despite escalating medical expenditures associated with arthritis treatment, patient quality of life has not substantially improved, with individuals enduring persistent pain, disability, and psychological stress [11,12]. Given the rapid aging of populations worldwide, reducing the burden of arthritis has emerged as a major public health priority. Investigating arthritis risk factors within this societal context holds profound implications for preventive strategies.

Agricultural work is a broad and complex field that encompasses multiple aspects, including agriculture, forestry, animal husbandry, fishing, and more involve physical activity behavior, which refers to any activity caused by skeletal muscle contraction. Methodical and purposeful physical exercise can reduce pain and improve function in patients with OA of the knee or hip [13,14], but at the same time physical activity leads to energy expenditure [15]. It is speculated that repetitive joint movements and joint loading involved in agricultural work exercise can accelerate joint degradation and cause OA [16]. Other study have noted that the prevalence of OA (osteoarthritis) is influenced by age, annual hours of work, and type of agriculture. In particular, it is important to note that there is a positive correlation between the incidence of OA in the thumb and long hours of work and specific types of agriculture [17]. In addition, Elizabeth M et al. conducted a study on labour force participation in patients with OA versus those without OA. The results showed that patients with OA were more likely to drop out of the labour market compared to patients without arthritis or without joint symptoms [18]. And the study by Eleuterio A et al. highlights that dysbiosis may lead to chronic inflammation, which may exacerbate arthritis in physically active agricultural workers [19]. Studies have shown that in specific subgroups of individuals with low lean mass index (LMI), certain weight-bearing activities may increase the risk of knee osteoarthritis (KOA) [20]. Repetitive stepping activities significantly increase medial acceleration in KOA patients [21]. The relationship between OA and the overuse and load-bearing of joints suggests that similar joint burdens in agricultural labor may be related to the development of OA [22]. However, there are not currently studies directly linking agricultural labor to an increased risk of OA. Therefore, it is necessary to explore in more detail the association between agricultural labor and the development of arthritis. Compared with previous studies, this study used CHARLS data and, secondly, integrated multiple bioinformatics methods to delve into the intrinsic mechanism of the relationship between agricultural activities and arthritis, to explore the association between agricultural work and arthritis in Chinese people, and to provide ideas and strategies for early detection of arthritis.

China Health and Retirement Longitudinal Study (CHARLS) aims to collect a set of high-quality microblogs representing families and individuals aged 45 and above in China Observing data to analyze China’s aging population and promote interdisciplinary research on aging issues. Nowadays, the advantages of the CHARLS database in studying diseases are gradually increasing. Luo et al. used data from the CHARLS in 2018 to examine the association between depression and obesity in middle-aged and older Chinese men and women [23]. Hu et al. conducted a cross-sectional analysis in 2015 to investigate the association between sarcopenia and cognitive function; Based on 2018 data, further analysis is conducted on the longitudinal association between sarcopenia and cognitive impairment in elderly people aged 60 and above in China, aiming to provide objective scientific evidence for the etiology, early intervention, and prevention strategies of cognitive impairment [24].

This study is based on the CHARLS database and uses multiple statistical methods to analyze the impact of various confounding factors and agricultural work on arthritis, ultimately identifying the risk factors that affect arthritis and providing reference for the improvement and prevention of arthritis.

## 2. Materials and methods

### 2.1. Data source

This is an observational study, A CHARLS (http://charls.pku.edu.cn/), which focuses on tracking the health and aging of the Chinese population, was used to collect high-quality microdata representing households and individuals aged 45 and above in China to analyze the issue of population aging. Informed written consent was provided by all participants. The CHARLS sampling aimed to ensure that the samples were unbiased and representative. It was carried out in four stages at the county (district), village (residential area), household and individual levels. At the county (district) and village (residential area) levels, probability – proportional – to – size sampling (PPS sampling) was adopted. At the county level, based on the population in 2009, using region, urban – rural areas and GDP as stratification indicators, 150 counties or districts were randomly selected from 30 provincial – level administrative units (excluding Tibet, Taiwan, Hong Kong and Macao). At the village level, based on the permanent population in 2009, three villages or communities were randomly selected from each of these 150 counties or districts, resulting in a total of 450 villages or communities.

**Fig 1:**
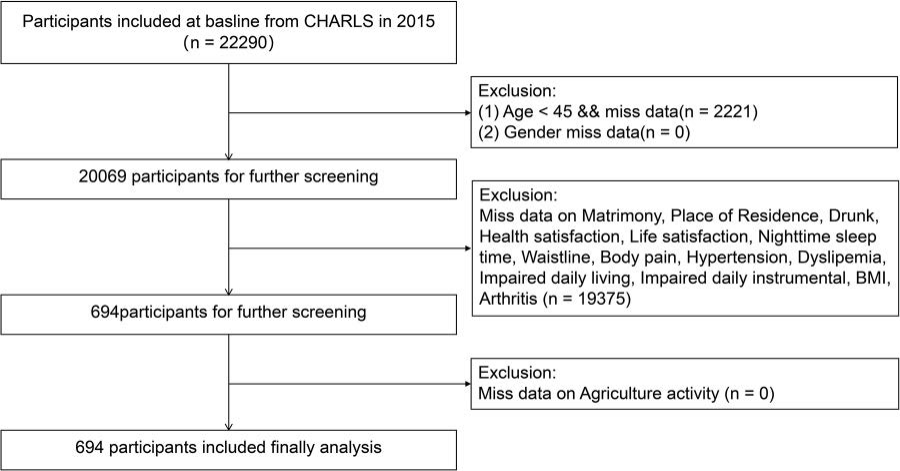
Recruitment process for 694 participants.

In this analysis, 22,290 participants from the year 2015 were initially included, with individuals under 45 years old and those with missing age data being excluded. Additionally, participants lacking other covariate data (gender, matrimony, place of residence, drunk, health satisfaction, life satisfaction, nighttime sleep time, waistline, body pain, hypertension, dyslipemia, impaired daily living, impaired daily instrumental, body mass index (BMI) and agricultural work) were also excluded, resulting in the recruitment of 694 participants (Fig 1).

### 2.2. Definition of variable

The outcome of this study, arthritis, was defined based on the questionnaire question “Have you been diagnosed with arthritis by a doctor?”, with those answering yes defined as the disease group and those answering no defined as the control group. The exposure factor in this study, agriculture activity, was defined based on the questionnaire question “Engage in agriculture activity for more than 10 days?”, with those answering yes considered as engaged and those answering no considered as not engaged. In addition, several important covariates (age, gender, matrimony, place of residence, drunk, health satisfaction, life satisfaction, nighttime sleep time, waistline, body pain, hypertension, dyslipemia, impaired daily living, impaired daily instrumental, body mass index (BMI)) were selected (Table 1).

**Table 1:** Information on questionnaire issues.

Variable	Year	Categories
Age	2015	45-54 years, 54–64 years, 64–74 years, over 74 years
Gender	2015	Male, Female
Marital Status	2015	Married, Separated, Divorced, Widowed, Unmarried, Cohabitated
Place of Residence	2015	Town, Village
Alcohol Consumption	2015	Yes, No
Health Satisfaction	2015	Completely, Very, Somewhat, Not very, Not at all
Life Satisfaction	2015	Completely, Very, Somewhat, Not very, Not at all
Nighttime Sleep Time	2015	Continuous variable
Waist Circumference	2015	Continuous variable
Body Pain	2015	Yes, No
Hypertension	2015	Yes, No (Systolic blood pressure ≥ 140 mmHg or Diastolic blood pressure ≥ 90 mmHg)
Dyslipidemia	2015	Yes, No (Total cholesterol ≥ 240 mg/dL or HDL ≤ 40 mg/dL or LDL ≥ 160 mg/dL or triglyceride ≥ 150 mg/dL)
Impaired Daily Living	2015	Yes, No (Any difficulty or inability in activities like dressing, bathing, eating, getting out of bed, using the toilet, and defecating)
Impaired Daily Instrumental Activities	2015	Yes, No (Any difficulty or inability in activities like doing housework, preparing hot meals, shopping, making phone calls, taking medication, and managing finances)
BMI	2015	underweight (BMI < 18.5 kg/m^2^), normal (BMI = 18.5 kg/m^2^ ~ 24 kg/m^2^), overweight (BMI ≥ 24 kg/m^2^)
Arthritis	2015	Yes, No
Agriculture_activity	2015	Yes, No

### 2.3. Statistical analysis

In this study, based on baseline characteristics, categorical variables were expressed as percentages, and continuous variables were presented as weighted means ± standard deviation (SD). Student t-tests and chi-square tests were utilized for the differences analysis of categorical and continuous variables (*P* < 0.05). In order to examine the relationship between engaging in agriculture activity and the presence of arthritis, three multivariable glm regression models were developed. In the regression analysis, all confounding factors were incorporated into the model to evaluate the relationship between the exposure factors and the outcomes. Then, the data were stratified according to the confounding factors, and the exposure-outcome relationships in each stratum were evaluated respectively. And the multivariate regression model could help researchers control confounding factors, conduct stratified analysis, predict and interpret results, and evaluate the fitting effect of the model, thus providing strong statistical support for relevant research.

Model 1 was unadjusted for any covariates except agriculture activity. Model 2 included adjustments for age and gender (Age: Age is an important risk factor for the development of arthritis, and the risk of arthritis increases with age. Gender: Most types of arthritis are more common in women, so gender is an important confounding factor.) in addition to the variables in model 1. Model 3 further adjusted for matrimony, place of residence, drunk, health satisfaction, life satisfaction, nighttime sleep time, waistline, body pain, hypertension, dyslipemia, impaired daily living, impaired daily instrumental, and BMI (Marital status: Marital status may influence an individual’s lifestyle and health behaviors, which may affect the risk of arthritis. Place of residence: Living in a rural or urban area may affect an individual’s health status and lifestyle. Alcohol consumption: Alcohol consumption may be associated with a variety of health problems, including the development of arthritis. Health Satisfaction and Life Satisfaction: These factors may affect an individual’s overall health and perceived behavior. Nighttime sleep duration: Sleep deprivation may increase the risk of multiple health problems, including arthritis. Waist size and body pain: obesity and body pain are both known risk factors for the development of arthritis. High blood pressure and high blood cholesterol: these metabolic disorders have been linked to the development of arthritis. Limitations in daily activities and limited use of everyday tools: these indicators reflect an individual’s state of physical functioning and may influence the development of arthritis. BMI: overweight and obesity are important risk factors for arthritis.) on top of the variables in model 2. Weighted logistic regression analysis was then conducted to explore the stability of the relationship between agriculture activity and arthritis across different populations.

The diagnostic capability of agriculture activity for arthritis was assessed by plotting a receiver operating characteristic (ROC) curve using the pROC package (v 1.18.0) [25] with an area under the curve (AUC) > 0.7. To assess the clinical utility of different models, decision curve analysis (DCA) was performed using the rmda function of ggDCA package (v1.1).

Furthermore, the net reclassification index (NRI) was employed to evaluate the improvement in predictive accuracy of the new model compared to the old model, with positive values indicating improvement. The integrated discrimination improvement (IDI) was used to reflect changes in the predicted probability gap between the two models, with positive values suggesting enhanced predictive ability of the new model. Statistical analyses in this study were conducted using R packages, with a significance level set at *P* < 0.05.

### 2.4. Ethics approval and consent to participate

Not applicable.

## 3. Results

### 3.1. Five covariates had an effect on arthritis

Two groups were divided based on whether they were diagnosed with arthritis, with 443 samples as controls and 251 samples as diseased. As shown in the baseline table, five covariates, namely health satisfaction (*P* = 0.002), life satisfaction (*P* = 0.004), nighttime sleep time (*P* < 0.001), body pain (*P* < 0.001), and agriculture activity (*P* = 0.026), were found to have a significant impact on arthritis (Table 2).

**Table 2:** Results for baseline table.

	Level	Control	Disease	p^a^
n		443	251	
Age (%)	45-54_year	179 (40.4)	79 (31.5)	0.07
	54-64_year	115 (26.0)	74 (29.5)	
	64-74_year	92 (20.8)	68 (27.1)	
	Over_74_year	57 (12.9)	30 (12.0)	
Gender (%)	Female	272 (61.4)	160 (63.7)	0.595
	Male	171 (38.6)	91 (36.3)	
Matrimony (%)	Divorced	4 (0.9)	2 (0.8)	0.794
	Married	387 (87.4)	217 (86.5)	
	Separated	3 (0.7)	1 (0.4)	
	Un_married	7 (1.6)	2 (0.8)	
	Widowed	42 (9.5)	29 (11.6)	
Place_of_Residence (%)	Town	131 (29.6)	69 (27.5)	0.621
	Village	312 (70.4)	182 (72.5)	
Drunk (%)	No	300 (67.7)	175 (69.7)	0.646
	Yes	143 (32.3)	76 (30.3)	
Health_satisfaction (%)	Completely	13 (2.9)	2 (0.8)	0.002
	Not_at_all	41 (9.3)	29 (11.6)	
	Not_very	95 (21.4)	70 (27.9)	
	Somewhat	208 (47.0)	126 (50.2)	
	Very	86 (19.4)	24 (9.6)	
Life_satisfaction (%)	Completely	26 (5.9)	14 (5.6)	0.004
	Not_at_all	5 (1.1)	9 (3.6)	
	Not_very	41 (9.3)	24 (9.6)	
	Somewhat	209 (47.2)	143 (57.0)	
	Very	162 (36.6)	61 (24.3)	
Nighttime_sleep_time (mean (SD^b^))		6.46 (1.86)	5.82 (2.11)	<0.001
Waistline (mean (SD))		86.59 (11.59)	85.19 (14.82)	0.171
Body_pain (%)	No	286 (64.6)	114 (45.4)	<0.001
	Yes	157 (35.4)	137 (54.6)	
Hypertension (%)	No	299 (67.5)	182 (72.5)	0.197
	Yes	144 (32.5)	69 (27.5)	
Dyslipemia (%)	No	292 (65.9)	155 (61.8)	0.309
	Yes	151 (34.1)	96 (38.2)	
Impaired_daily_living (%)	No	421 (95.0)	229 (91.2)	0.07
	Yes	22 (5.0)	22 (8.8)	
Impaired_daily_instrumental (%)	No	364 (82.2)	191 (76.1)	0.069
	Yes	79 (17.8)	60 (23.9)	
BMI (%)	Normal	194 (43.8)	119 (47.4)	0.58
	Overweight	230 (51.9)	120 (47.8)	
	Underweight	19 (4.3)	12 (4.8)	
Agriculture_activity (%)	No	231 (52.1)	108 (43.0)	0.026
	Yes	212 (47.9)	143 (57.0)	

^a^ p: p-value.

^b^ SD: standard deviation.

### 3.2. Significant positive association between agriculture activity and arthritis risk

**Fig 2:**
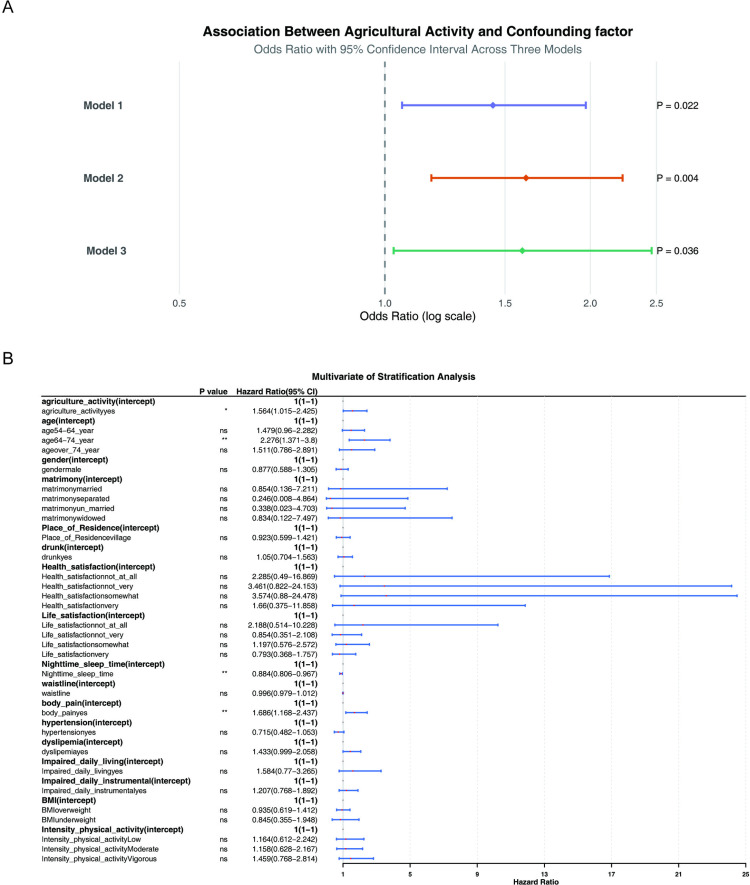
Results of risk stratification analysis.

In the constructed multiple variable glm regression models (Table 3), the positive association between agriculture activity and arthritis was found to be significant across all three models (model 1: odds ratios (OR) = 1.44, 95% confidence intervals (95% CI): 1.06–1.97, *P* = 0.021, ARD = 0.098; model 2: OR = 1.61, 95% CI: 1.17–2.24, *P* = 0.004, ARD = 0.121; model 3: OR = 1.74, 95% CI: 1.17–2.60, *P* = 0.007, ARD = 0.136). Furthermore, the *P* values for agriculture activity in all three models were less than 0.05, indicating that the impact of agriculture activity on arthritis was not significantly influenced by other covariates. Subsequent risk stratification analysis revealed that the effect of agriculture activity on arthritis remained undisturbed by other covariates, with agriculture activity being identified as a risk factor for arthritis (OR = 1.736, 95% CI: 1.168–2.597, *P* < 0.01) (Fig 2).

**Table 3:** Multivariate glm regression model constructed.

Exposure factor	Model 1_OR^a^ (95%_CI^b^)	Model 2_OR(95%_CI)	Model 3_OR (95%_CI)
Agriculture_activity_yes	1.44e + 00 (1.06e + 00-1.97e + 00)	1.61e + 00 (1.17e + 00-2.24e + 00)	1.74e + 00 (1.17e + 00-2.60e + 00)
p_value	0.02120	0.00393	0.00676

^a^ OR: odds ratio.

^b^ CI: confidence interval.

### 3.3. Perfect predictive performance of agriculture activity on arthritis

**Fig 3:**
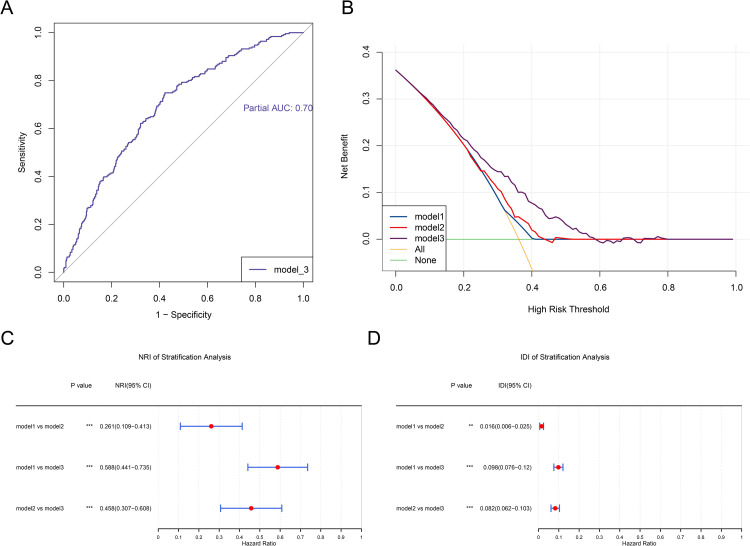
Assessment of the predictive performance of agricultural activities on arthritis. **(A)** Results of ROC curve analysis. **(B)** Results of decision curve analysis. **(C)** NRI stratification analysis of different models. **(D)** IDI stratification analysis of different models. ROC, receiver operating characteristic; NRI, net reclassification index; IDI, integrated discrimination improvement.

The ROC curve analysis was conducted to assess the predictive performance of agriculture activity on arthritis. The ROC curve analysis revealed that agriculture activity had an excellent predictive value for arthritis, with an AUC of 0.7 (Fig 3A). In the decision curve, the net benefit of model 3 was higher than that of model 2 and model 1, indicating that its performance improved after adjusting for multiple confounders compared to the unadjusted models (Fig 3B). Additionally, a comparison of the predictive accuracy and probability between different models was carried out. The forest plot demonstrated that the NRI and IDI for model 1 vs. model 2 (IDI = 0.016, 95% CI: 0.006–0.025; NRI = 0.261, 95% CI: 0.109–0.413), model 1 vs. model 3 (IDI = 0.098, 95% CI: 0.076–0.12; NRI = 0.588, 95% CI: 0.441–0.735), and model 2 vs. model 3 (IDI = 0.082, 95% CI: 0.062–0.103; NRI = 0.458, 95% CI: 0.307–0.608) were all greater than 0, suggesting that the predictive performance of model 3 was superior to that of model 1 and model 2 (Fig 3C, 3D).

## 4. Discussion

Arthritis is a common chronic disease that is mainly characterised by inflammation of the joints. Inflammation can lead to symptoms such as joint pain, swelling, stiffness, and limited movement [1–4]. And studies have shown that there are currently more than 100 million people with arthritis in China, with the number of people aged 50 years and older suffering from osteoarthritis steadily increasing [7,9]. Although the cost of arthritis treatment is increasing, patients have no substantial improvement in their quality of life and have to endure continuous pain, disability and psychological stress [11,12]. Therefore, reducing the burden of arthritis is an important public health task in the context of a rapidly ageing global population. In this study, we analysed the effects of confounding factors and agricultural work on arthritis using multiple statistical methods based on the CHARLS database and found a significant correlation between agricultural work and arthritis, which informs arthritis improvement and prevention.

The relationship between agricultural activities and human disease has been the focus of research [26], particularly the health effects of physical activity in agricultural settings. The study has found that people living in rural areas or engaged in agricultural activities had a higher incidence of arthritis due to long-term physical work, especially repetitive and heavy physical work [27,28]. This study found that agricultural activities significantly increased the risk of arthritis. The risk stratification analysis further showed that the association remained significant when controlled for different covariates, suggesting a closer relationship between agricultural activity and arthritis. Mechanical stress and joint load in agricultural activities are important factors leading to joint degradation and inflammation [29]. OA is thought to be the result of an interaction between systemic susceptibility and abnormal body stress, which may result from overloading or malformation of the joints [30]. Mechanical overload damages biochemical pathways in chondrocytes, leading to reduced production of extracellular matrix (ECM) and increased degradation of ECM molecules by related proteases in chondrocytes [31]. Iron death is a recently discovered pathophysiological form of cell death that can lead to mitochondrial dysfunction and cellular oxidative damage [32]. Iron death occurred in chondrocytes in the articular cartilage load zone and in chondrocytes stimulated by mechanical overload in patients with OA, consistent with chondrocyte degeneration. This suggests that mechanical load-related chondrocyte iron death may be involved in the aging process of cartilage [33]. Chondrocyte death plays a key role in the pathogenesis of OA [34]. The normal healing process that occurs after microinjury to the tendon site caused by mechanical stress is hijacked by disordered systemic immunity, resulting in persistent tendinitis and abnormal tissue healing. In RA, the result is bone erosion, probably due to long-term activation of osteoclasts in the subchondral bone near the tendon attachment site [35]. In addition, people engaged in agricultural activities, especially workers who need to get up early and work long hours, tend to sleep less. This lack of sleep can lead to inadequate physical recovery, increased joint burden, and an increased risk of arthritis. Good sleep habits are also important for the joint health of agricultural workers [36]. Agricultural workers may experience metabolic abnormalities such as insulin resistance and hypertriglyceridemia due to high-intensity physical labor. These metabolic abnormalities are associated with increased TyG index. Studies have shown that WC*TyG reflects the hypertriglyceridemia phenotype, indicating that it can be used as a good indicator to define the metabolic syndrome phenotype. The higher the WC*TyG, the higher the prevalence of MetS, and the increased TyG index is closely related to the increased risk of arthritis [37]. Agricultural workers need to pay attention to their metabolic health and reduce the risk of arthritis through a reasonable diet and appropriate physical activity [38]. In addition, some studies have shown an association between the gut microbiome and lifestyle interventions in chronic pain patients, suggesting that lifestyle, such as diet, may increase arthritis risk [39]. Therefore, this study found that further attention to the health of agricultural populations may be beneficial for protection against arthritis.

Physical labor associated with agricultural activities significantly affects joint health. Long-term repetitive physical labor, insufficient sleep, and metabolic abnormalities are important factors in the onset of arthritis. In addition, the incidence of arthritis varies significantly by region, age, and gender [40]. From 1990 to 2019, the incidence of arthritis gradually increased. According to the 2019 Global Burden of Disease Study, the overall incidence of OA ranks 17th out of 369 diseases and injuries, and is more common in socioeconomically developed regions [41]. It is worth noting that in countries with higher sociodemographic indices, most patients have mild OA, while in countries with lower sociodemographic indices, most patients have moderate to severe OA [42]. The incidence of arthritis was also significantly associated with increasing age. Studies have found that patients before age of 30 have no disease burden. This may be because young people are less prone to muscle weakness and degeneration. In addition, senescent cells produced during the body’s natural aging process also play a role in the age-related manifestations of arthritis [43]. Arthritis also affects women more than men, and women tend to have more severe disease (i.e., structural lesions and clinical symptoms). This difference could be due to a number of reasons. Studies have found that there are significant differences in joint kinematics between men and women, which makes women more vulnerable [44]. Women’s weaker muscle strength may also contribute to gender differences [45]. This is consistent with our findings that the risk of arthritis in agricultural activities is different in different age groups, different genders, and different regions.

These studies reveal the health challenges faced by agricultural workers and provide scientific basis for developing health management strategies. By improving working conditions and lifestyle habits, agricultural workers can effectively reduce disease risks and improve their quality of life. In addition, studies have shown that the use of care platforms can impact the surgical experience by increasing patient engagement, facilitating remote monitoring and providing personalised care [46]. In addition to the impact of exposure factors on arthritis, related studies have also shown that intermediates such as lactic acid have a close relationship with arthritis. Joint inflammation in RA patients is believed to occur in hypoxic microenvironments, leading to imbalanced lactic acid metabolism and accumulation. Lactic acid is no longer considered merely a metabolic waste product of glycolysis but a promoter of RA development [47]. Furthermore, studies have found that lactic acid accumulation helps human CD4 + T cells upregulate the lactic acid transporter SLC5A12, affecting their differentiation, activation, and function, thereby accelerating the development of RA [48]. Ectopic lymphoid structures (ELS) that develop in inflammatory tissue may play a key pathogenic role in autoimmunity and serve as potential biomarkers for disease development and therapeutic response, which is gaining attention [49–51]. It was found that human CD4 + T cells up-regulated the expression of lactate transporter SLC5A12 in human RA synovium. And surprisingly, its levels were significantly correlated with RA synovial tissue T cell scores and the formation of IL17-rich ELS [52–55], thus suggesting that lactic acid/SLC5A12-induced metabolic signaling may play a role in promoting chronic inflammation in RA. Therefore, excessive agricultural labor may cause lactic acid metabolism to be too fast or abnormal, thus promoting the development of arthritis.

Using the CHARLS database, this study fills the gap in research on the association between agricultural activities and arthritis. CHARLS provides rich demographic, health status, and lifestyle data, enabling detailed multivariate analysis. It helps health policy makers and public health practitioners to develop more targeted arthritis prevention measures for agricultural workers.

Although the research findings contribute to understanding the relationship between agricultural activities and arthritis, they still have certain limitations. Firstly, the data only comes from the Chinese population, which means that the applicability of the research results to other populations may be limited. Secondly, the study did not analyze the different subtypes of arthritis, as arthritis itself is a complex disease that may include multiple types such as OA and RA, each with different etiologies and risk factors. In addition, there are some potential biases in this study, for example, selection bias may be caused by a lack of representativeness of the sample or bias in the recruitment process, while measurement bias may arise due to a lack of accuracy in the questionnaire design, bias in participant recall, or errors in the data collection session.

In the future, more confounding factors related to the link between agricultural activities and arthritis will be explored, like environmental factors (e.g., climate, geographic conditions), individual genetic factors, and lifestyle factors (e.g., diet, exercise habits). In addition, it has been shown that repetitive strain injuries lead to increased pain sensitivity over time, which may be similar to joint strain injuries caused by prolonged agricultural labour [56]. Therefore, the specific contribution of different types of agricultural activities to arthritis risk will be investigated in the future, e.g., the different effects of prolonged physical labour, specific agricultural handling positions, exposure to certain agricultural chemicals, etc. This has important theoretical and practical implications. Theoretically, it contributes to a deeper understanding of the complex factors that influence arthritis occurrence; in practice, the findings can guide healthcare providers to develop more effective early arthritis screening and prevention programmes for agricultural workers, e.g., tailoring workplace interventions (improving the working environment, providing appropriate rest breaks, promoting correct working posture, etc.) according to the risk characteristics of the different agricultural activities in order to reduce the incidence of arthritis, and Health education can be provided to agricultural workers to raise their awareness of arthritis risks and promote self-protection.

## 5. Conclusion

This study found an association between agricultural activity and arthritis risk, baseline statistics showed significant population size differences between those with the disease who worked in agriculture and controls, correlation analyses showed that the effect of agricultural work on arthritis differed significantly among the three models and that the effect of confounders was small, and ROC validation showed that among the three models model 3 was the stronger predictor of arthritis and had the highest net intervention benefit over risk, and the highest benefit of model 3 indicated that adjusting the model with multiple confounders was better than the single feature model, in addition, NRI and IDI analyses also showed that model 3 was better than model 1. In summary, the present study analysed the effects of confounders and agricultural work on arthritis using multiple statistical methods thus confirming that agricultural work can predict the occurrence of arthritis, and provide ideas for the early detection and prevention of arthritis. This study provides ideas for early detection and prevention of arthritis.

## Supporting information

S1 Fig Forest map of the association between agricultural activities and the severity of OA. The vertical line in the center is the null line, i.e., OR = 1, indicating that the study factors are not statistically significantly associated with the outcome. The blue dots indicate OR point estimates, and the horizontal line where the blue dots are located indicates the 95% confidence interval for the OR value; when the horizontal line is to the right of the null line, it indicates that the study factor is positively associated with the occurrence of the outcome event.
(TIF)

S1 Table OA Severity. (TIF)

S2 Table Association analysis table of exposure factors and outcomes. (TIF)
