# Supplementary figures and images for: The association between agricultural activities and arthritis in middle-aged and elderly people: Findings from a cohort study based on CHARLS
Source: PLoS One. 2025 Jun 26;20(6):e0326447. doi: 10.1371/journal.pone.0326447 (PMC12200726; doi:10.1371/journal.pone.0326447)

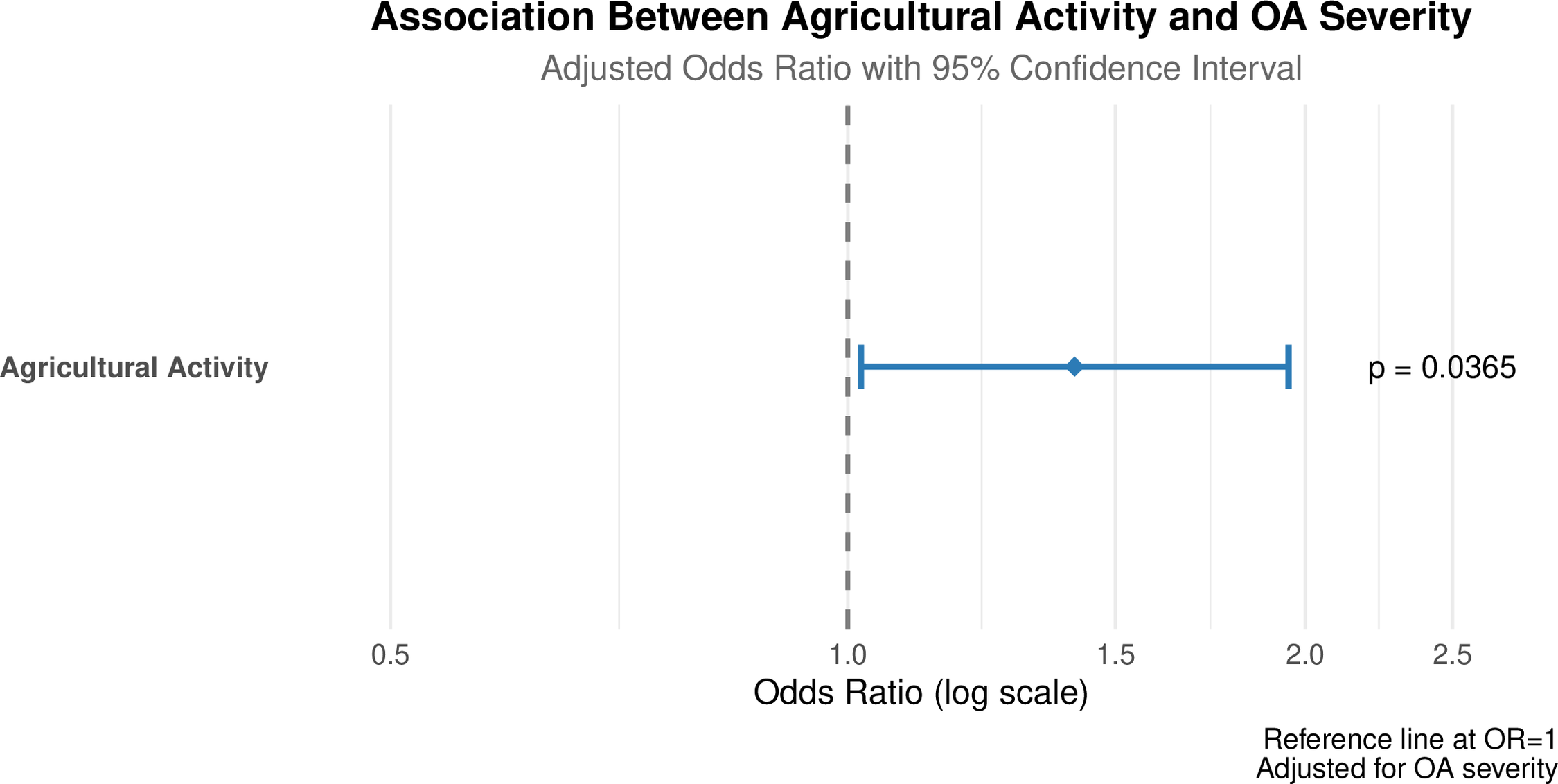

Supplement: S1 Fig — The vertical line in the center is the null line, i.e., OR = 1, indicating that the study factors are not statistically significantly associated with the outcome. The blue dots indicate OR point estimates, and the horizontal line where the blue dots are located indicates the 95% confidence interval for the OR value; when the horizontal line is to the right of the null line, it indicates that the study factor is positively associated with the occurrence of the outcome event. (TIF) [file pone.0326447.s001.tif]

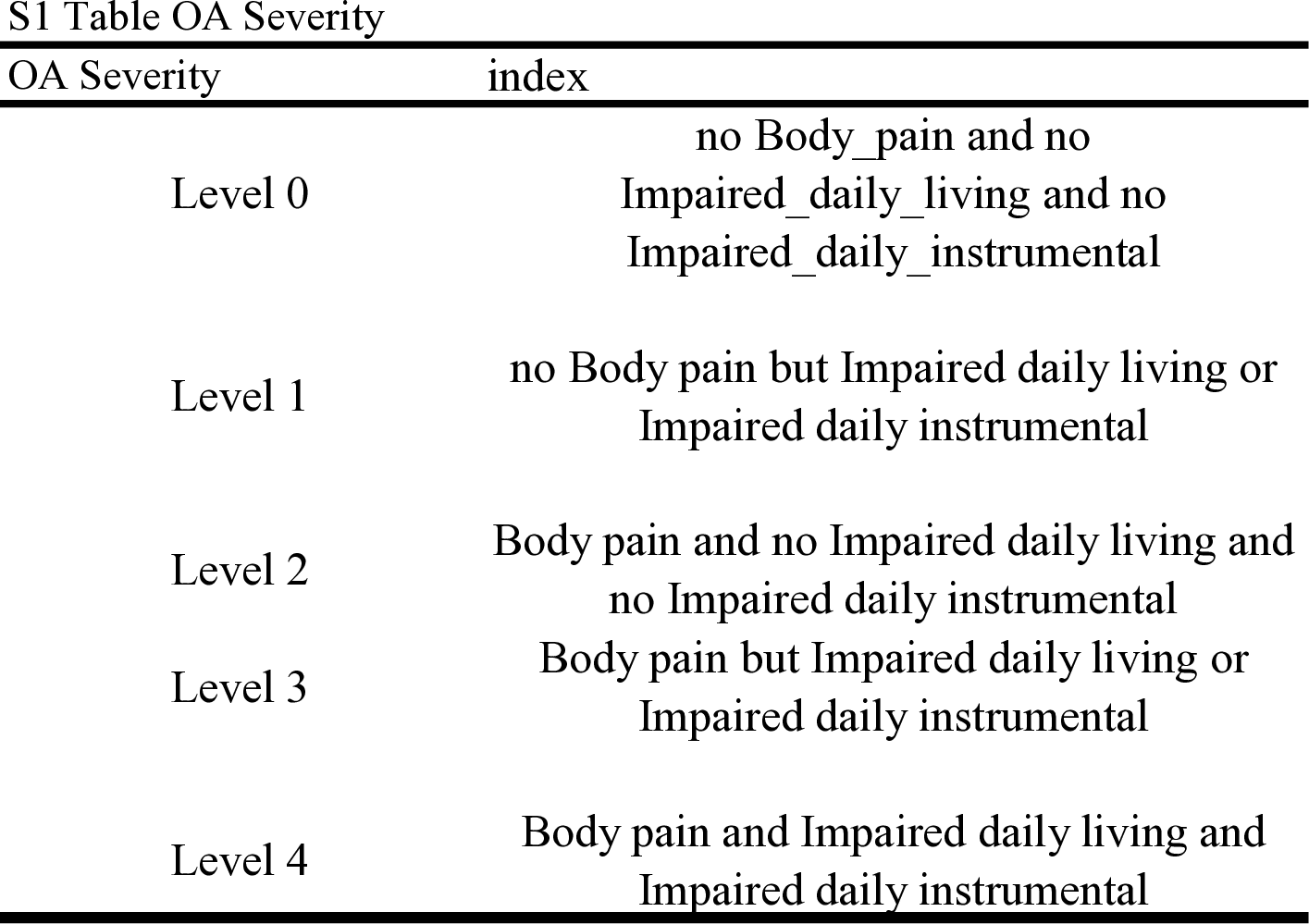

Supplement: S1 Table — (TIF) [file pone.0326447.s002.tif]

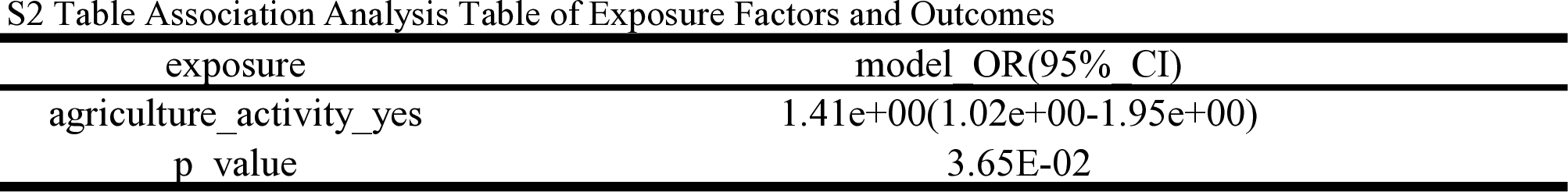

Supplement: S2 Table — (TIF) [file pone.0326447.s003.tif]
